# Mixed lymphocytic and collagenous inflammation of the entire gastrointestinal tract under therapy with serotonin and norepinephrine reuptake inhibitors

**DOI:** 10.1007/s00428-022-03351-2

**Published:** 2022-06-02

**Authors:** Ana I. Varelas, Stefan Fürst, Cord Langner

**Affiliations:** 1 Department of Pathology, Portuguese Institute of Oncology, Porto, Portugal; 2 Diagnostic and Research Institute of Pathology, Diagnostic and Research Centre for Molecular BioMedicine, Medical University of Graz, Neue Stiftingtalstraße 6, 8010 Graz, Austria; 3 Department of Internal Medicine, Division of Gastroenterology and Hepatology, Medical University of Graz, Graz, Austria

**Keywords:** Drug-induced injury, Lymphocytic esophagitis, Collagenous gastritis, Lymphocytic duodenitis, Collagenous colitis, Microscopic colitis

## Abstract

Drug-induced injury to the gastrointestinal tract has gained growing significance in recent years, and the list of causative medications keeps expanding. Herein, we present the case of a 45-year-old female with major depressive disorder treated with two serotonin and norepinephrine reuptake inhibitors (venlafaxine and duloxetine). She developed nausea and weight loss. Endoscopic evaluation of the upper and lower gastrointestinal tract rendered grossly normal mucosa in all segments. Histological examination, however, revealed lymphocytic esophagitis, collagenous gastritis, celiac disease-like intraepithelial lymphocytosis of the duodenum, and incomplete collagenous colitis. Gastrointestinal side effects of psychoactive drugs are largely underrecognized. This is the first report of a mixed lymphocytic and collagenous pattern of injury affecting esophagus, stomach, duodenum, and colon triggered by combined treatment with venlafaxine and duloxetine. In patients with unclear symptoms, obtaining biopsies from mucosa that is normal upon endoscopic inspection may render decisive clues for clinical management.

## Introduction

With the advent of more selective and targeted treatments, drug-induced injury to the gastrointestinal tract has gained growing significance, while awareness is staying behind. Side effects may be encountered in virtually every part of the gastrointestinal tract with different histological patterns of involvement. While some drugs predominantly affect the upper tract, such as iron pills, proton pump inhibitors (PPIs), and angiotensin II receptor blockers, others mainly involve the lower tract, such as mycophenolate mofetil and immune checkpoint inhibitors [1, 2].

The gastrointestinal side effects of psychoactive drugs are underrecognized. Selective serotonin reuptake inhibitors (SSRIs), which are the most commonly prescribed antidepressants, have been associated with the development of microscopic colitis, that is, lymphocytic and collagenous colitis [3, 4]. The gastrointestinal toxicity caused by other antidepressants is less well established.

Herein, we report the histological findings that occurred under combined therapy with two serotonin and norepinephrine reuptake inhibitors (SNRIs), a class of drugs that is likewise effective in treating depression. All segments of the gastrointestinal tract were found to be affected, showing lymphocytic, collagenous, or mixed lymphocytic and collagenous inflammation.

## Case report

A 45-year-old female under combined therapy with venlafaxine and duloxetine for major depressive disorder presented with nausea and weight loss, no diarrhea. Laboratory tests were unremarkable, specifically celiac disease serology was negative.

**Fig. 1:**
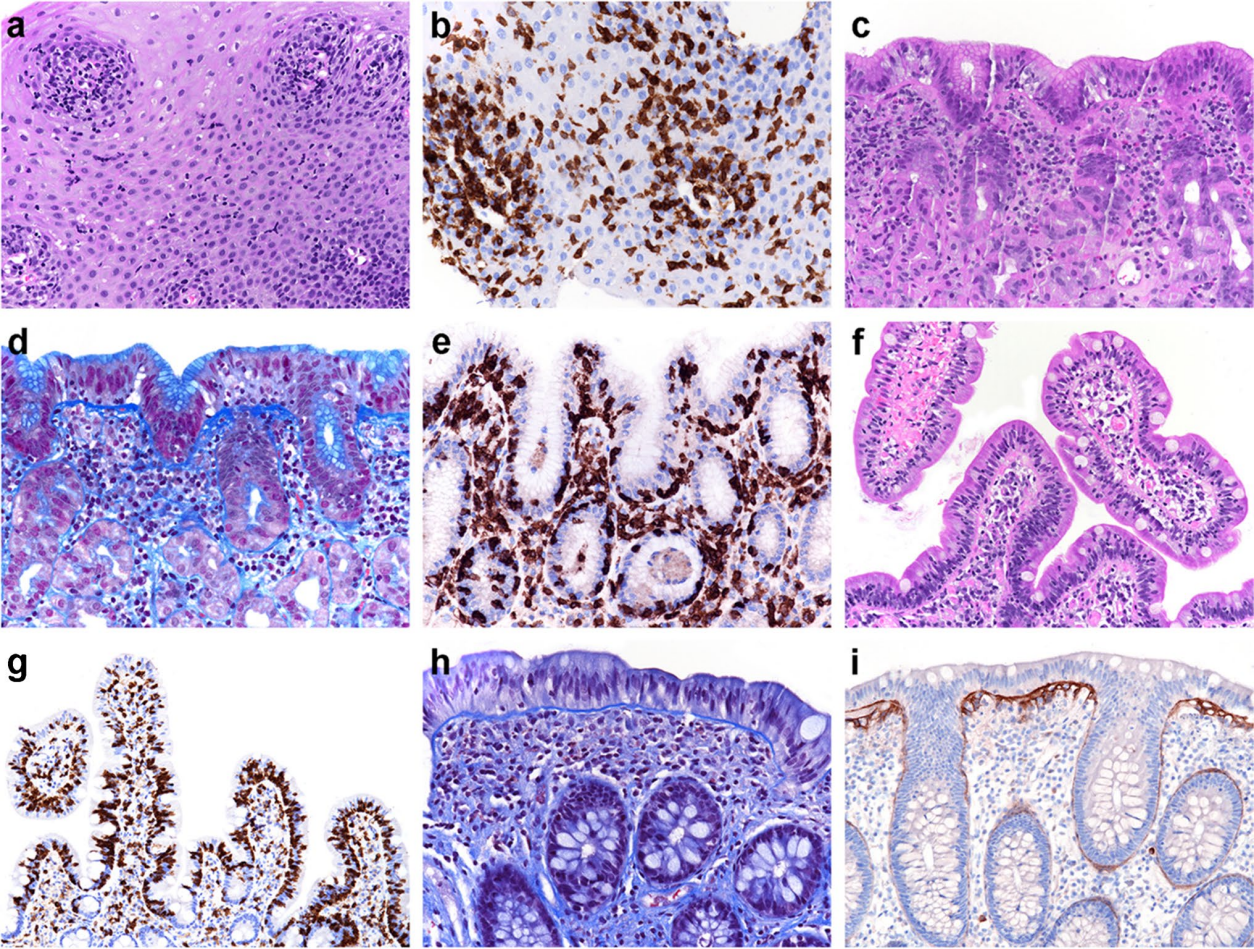
The biopsy material from the esophagus shows a lymphocytic esophagitis pattern of injury with more than 40 lymphocytes per high power field (**A**, hematoxylin and eosin (H&E) stain; **B**, CD3 immunohistochemistry). Mild chronic inactive inflammation is observed in the stomach with thickening of the subepithelial collagen band, consistent with diagnosis of collagenous gastritis (**C**, H&E stain; **D**, chromotrope aniline blue (CAB) stain); intraepithelial lymphocytes are mildly increased (**E**, CD3 immunohistochemistry). The biopsy material from the duodenum shows celiac disease-like morphology with > 60 intraepithelial lymphocytes per 100 epithelial cells, yet normal villous architecture (**F**, H&E stain; **G**, CD3 immunohistochemistry). There is an increased cell content within the lamina propria of the colon mucosa with mild thickening of the subepithelial collagen band, consistent with diagnosis of incomplete collagenous colitis (**H**, CAB stain; **I**, tenascin immunohistochemistry)

Gastroscopy was performed, and biopsies were taken from normal-looking esophageal, gastric, and duodenal mucosa. Histology revealed lymphocytic esophagitis (> 40 lymphocytes per high power field; Fig. 1A–B). A periodic acid–Schiff (PAS) stain performed to detect fungal organisms rendered a negative result. The mucosa of the gastric corpus showed mild chronic inactive inflammation with mild increase of intraepithelial lymphocytes (< 20 per 100 epithelial cells), yet significant thickening of the subepithelial collagen band (> 10 µ), as nicely illustrated by chromotrope aniline blue (CAB) stain (Fig. 1C–E). An additional tenascin immunostain was performed which highlighted the subepithelial deposits (not shown), ultimately leading to a diagnosis of collagenous gastritis. The biopsies obtained from the duodenum demonstrated a celiac disease-like morphology with significant increase of intraepithelial lymphocytes (> 60 per 100 epithelial cells), yet normal villous and crypt architecture (Fig. 1F–G).

The findings within the upper gastrointestinal tract prompted subsequent ileocolonoscopy. The mucosa was again normal upon endoscopic inspection. Step biopsies were taken. While the ileum was normal histologically, the colon mucosa showed a mild increase of cell content within the lamina propria with overrepresentation of eosinophils, in conjunction with few intraepithelial lymphocytes and mild thickening of the subepithelial collagen band (5–10 µ; positive for tenascin), consistent with a diagnosis of incomplete collagenous colitis (Fig. 1H–I).

## Discussion

Lymphocyte-rich inflammation of the esophagus, which has been reported as lymphocytic esophagitis (lymphocytic esophagitis-like pattern of injury), lichenoid esophagitis, or, more recently, lymphocyte-predominant esophagitis [5], represents a characteristic, yet not specific morphological pattern that may be observed in association with immune-mediated and motility disorders or secondary to drug use. A PAS stain should always be performed to exclude infection, particularly fungal infection, ideally on esophageal smears, that is, applying brush cytology.

Collagenous gastritis is a rare condition that is characterized by lamina propria inflammation, subepithelial collagen deposition, and potentially surface epithelial damage. The disorder may affect different areas of the stomach in both children and adults. Some patients have associated celiac disease, collagenous sprue, or collagenous colitis [6, 7]. In one study, the majority of adult patients were identified to take prescribed medication with 8 of 17 (47%) patients on five or more drugs [7]. Of note, 6 (35%) patients were on antidepressants, with two of them taking venlafaxine. Five (29%) patients were on olmesartan (angiotensin II receptor blocker); two of these had duodenal intraepithelial lymphocytosis with negative celiac disease serology [7].

Several drugs have been reported to induce a celiac disease-like morphology within the small bowel with prominent intraepithelial lymphocytosis and varying degree of architectural abnormalities, that is, crypt hyperplasia and villous atrophy [8]. These mainly include angiotensin II receptor blockers, such as olmesartan, but also temisartan, valsartan, and others, non-steroidal anti-inflammatory drugs (NSAIDs), and immune checkpoint inhibitors [8]. Morphological overlap with autoimmune enteropathy may occur. That disease often shows a complex histological picture, combining celiac disease-like and inflammatory bowel disease-like features with increased apoptosis.

The development of both lymphocytic and collagenous colitis has also been related to drug use [3, 4]. In particular, current exposure to NSAIDs, PPIs, or SSRIs and prolonged use for 4–12 months may increase the risk of developing microscopic colitis [3]. Of note, a subset of patients with olmesartan-induced enteropathy develops colon involvement in the form of microscopic colitis. Finally, a “lymphocytic colitis-like pattern of injury” has been reported for patients under immune checkpoint inhibitors or comparable biological agents, such as idelalisib.

Venlafaxine and duloxetine are SNRIs that have been approved for the treatment of major depressive and anxiety disorders and are sometimes also used to treat chronic pain, especially nerve pain. Seven clinical case reports are available on patients who developed gastrointestinal symptoms under treatment with venlafaxine and/or duloxetine and showed distinct morphological changes on biopsy diagnosis. All patients had microscopic colitis: four had lymphocytic colitis [9–12], two collagenous colitis [13, 14], and one patient presented with a mixed pattern [15]. One patient with collagenous colitis had synchronous collagenous ileitis [13]. Two of the seven patients had a celiac disease-like pattern in the duodenum [9, 14], one of which was described as “collagenous sprue” [14]. In the majority of patients, symptoms resolved within days or few weeks after drug withdrawal. Details are presented in Table 1. It is of note that follow-up biopsies to document histologic resolution for proof of concept have not been undertaken in any of these patients and were also not performed in our case yet.

**Table 1:** Published case reports on gastrointestinal injury associated with venlafaxine and/or duloxetine treatment (including the presented case at the bottom of the table)

Authors, year of publication	Age / gender	Relevant history and medication	Clinical complaints	Findings in the upper gastrointestinal tract	Findings in the lower gastrointestinal tract	Follow-up
Béchade et al., 2009 (ref #9)	67 / male	Established diagnosis of celiac disease (positive serology), depressive disorder treated with venlafaxine	Aggravation of chronic diarrhea after initiation of venlafaxine treatment, weight loss	Celiac disease (Marsh type III)	Lymphocytic colitis (with ileal involvement)	Remission of symptoms after termination of venlafaxine treatment
Kusnik and Stolte, 2010 (ref #10)	80 / female	Urinary incontinence treated with duloxetine	Watery diarrhea, weight loss	Normal duodenal biopsies	Lymphocytic colitis	Remission of symptoms after termination of duloxetine treatment
Gwillim and Bowyer, 2012 (ref #11)	50 / female	Depressive disorder treated with duloxetine	Watery diarrhea, abdominal pain, bloating	Upper GI endoscopy not performed	Lymphocytic colitis	Remission of symptoms after termination of duloxetine treatment
Sisman et al., 2012 (ref #12)	66 / female	Depressive disorder treated with duloxetine	Watery diarrhea, abdominal pain	Normal duodenal biopsies	Collagenous colitis (with ileal involvement)	Partial remission of symptoms after termination of duloxetine treatment (budesonide therapy initiated)
Bahin et al., 2013 (ref #13)	75 / female	Depressive disorder treated with venlafaxine, switched to duloxetine	Watery diarrhea, weight loss (after initiation of duloxetine treatment)	Normal duodenal biopsies	Mixed collagenous and lymphocytic colitis	Remission of symptoms after termination of duloxetine treatment
Yau et al., 2015 (ref #14)	56 / female	Depressive and anxiety disorder treated with duloxetine, switched to venlafaxine	Watery diarrhea, abdominal pain, bloating and flatulence	Collagenous sprue (Marsh type III), normal gastric mucosa	Collagenous colitis	Remission of symptoms after termination of duloxetine treatment, but recurrence of symptoms under treatment with venlafaxine (with persistence after termination)
Millán-Nohales et al., 2021 (ref #15)	26 / female	Borderline personality disorder and long-term bulimia nervosa treated with duloxetine	Watery diarrhea, abdominal pain	Normal duodenal biopsies	Lymphocytic colitis	Remission of symptoms after termination of duloxetine treatment
Presented new case	45 / female	Major depressive disorder, combined treatment with venlafaxine and duloxetine	Nausea and weight loss, no diarrhea	Lymphocytic esophagitis, collagenous gastritis, lymphocytic duodenitis	Incomplete collagenous colitis	No available information due to short-term follow-up

The presented case nicely illustrates that inflammatory changes suggestive of SNRI-related toxicity may be observed along the entire gastrointestinal tract, within every segment, from the esophagus to the colon, even within a single patient. It is of note for pathologists that the inflammation is primarily of “lymphocytic type,” i.e., lymphocytic esophagitis and lymphocytic duodenitis (celiac disease-like pattern), potentially leading to subepithelial collagen deposition, i.e., collagenous gastritis, collagenous duodenitis (sprue), and collagenous colitis.

In conclusion, gastrointestinal side effects of drugs represent a growing challenge for histopathologists. Specifically, SNRI-induced toxicity may be encountered along the entire gastrointestinal tract, showing lymphocytic, collagenous, or mixed lymphocytic and collagenous inflammation. Pathologists need to be aware of this peculiar morphological pattern. In patients with unclear symptoms, obtaining biopsies from mucosa that is normal upon endoscopic inspection may render decisive clues for clinical management, since cessation of treatment may lead to resolution of drug-induced symptoms.

## Data Availability

Data sharing not applicable to this article as no datasets were generated or analyzed during the current study.
